# Sciatic Hernia Mimicking Perianal Abscess in a Cirrhotic Patient

**DOI:** 10.1155/2012/651472

**Published:** 2012-10-18

**Authors:** Wellington Andraus, Luciana Bertocco de Paiva Haddad, Oscar Cavalcante Ferro, Luiz Augusto Carneiro D'Albuquerque

**Affiliations:** Gastroenterology Department, University of São Paulo School of Medicine, Rua Dr. Enéas de Carvalho Aguiar 255, 9° Andar, Sala 9113/9114, 05403-900 São Paulo, SP, Brazil

## Abstract

Abdominal hernias are very frequent in cirrhotic patients with ascites. The hernias usually present as umbilical, inguinal, incisional, or femoral. However, these patients can also develop uncommon hernias such as pelvic hernias because of pelvic floor weakness and high abdominal pressure due to ascites. We present the first case of a cirrhotic patient with ascites that developed a giant sciatic hernia mimicking a perianal abscess.

## 1. Introduction

Pelvic hernias are very rare, and sciatic is the rarest between them [1]. These hernias occur in the sciatic foramina formed by the sacrospinous ligament. The rarity makes the diagnosis a challenge and also increases the chance of a misdiagnosis. On the other hand, abdominal hernias are frequent in cirrhotic patients with ascites. However, these patients can also develop uncommon hernias such as pelvic hernias because of pelvic floor weakness and high abdominal pressure due to ascites [2]. 

The presence of advanced cirrhosis, with ascites, other abdominal hernias, and cirrhotic signs can confuse the physician and makes the diagnosis even more difficult. We present a cirrhotic patient with ascites that developed a giant sciatic hernia mimicking a perianal abscess that was misdiagnosed.

## 2. Case Description

**Figure 1:**
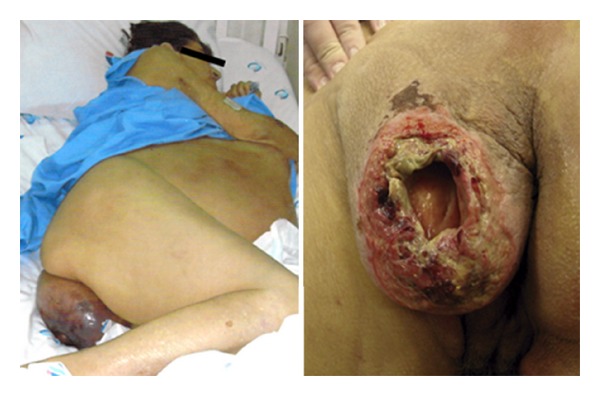
Hyperemic mass in the left perineum and left buttocks, that progressed to skin necrosis and perforation.

We present a case of a 64-year-old woman with a history of secondary biliary cirrhosis after a cholecystectomy. In addition to the cholecystectomy, the patient underwent two attempts to surgically repair a bile duct stenosis using enteric anastomosis. However, she progressed to advanced cirrhosis and reached a child C and MELD (model for end stage liver disease) score of 31. Three months prior to hospital admission, the patient presented with a tender, hyperemic mass in the left perineum and left buttocks and progressed to partial skin necrosis in this region (Figure 1). Other findings at the time of the physical examination included severe malnutrition, jaundice, anemia, ascites, and an epigastric hernia. She reported pain in the perineum region and developed renal insufficiency after hospitalization. The patient had no history of fever or changes in bowel movements. At the time of admission, the patient had a white cell count of 3 × 10^9^/L with a left shift, total bilirubin of 5.9 mg/dL, and an ascites culture positive for *Escherichia coli* and *Candida tropicalis*.

**Figure 2:**
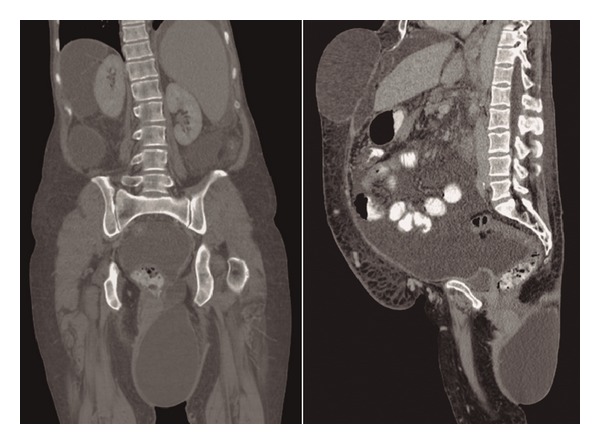
Computed tomography showing a sciatic hernia on the left side with a large hernial sac filled with liquid (ascites).

 Computed tomography showed a sciatic hernia (Figure 2) on the left side with a large hernial sac filled with liquid (ascites). Unfortunately, this patient was first seen in a small hospital a few days before admission to our hospital. Believing that it was a perianal abscess, she underwent a surgical drainage of the bulge, leaving an open hole in the hernial sac (Figure 1). Thereafter, the wound continually leaked ascites fluid, and secondary peritonitis developed. Her clinical condition consequently worsened. The misdiagnosis compromised the patient outcome. After admission, the patient required dialysis because of renal insufficiency. Her hepatic function worsened, and her MELD score reached 31. The patient underwent surgical repair of the hernia because there were no other options. The surgery consisted of a hernial sac dissection, placing sutures at its base. A polypropylene mesh plug was placed, and we used continuous suturing of several layers of tissue to promote impermeability. Postoperative ascites leakage was a concern, but this technique prevented its occurrence. Nevertheless, the patient was discharged from the intensive care unit, but she returned 3 days later because of a pulmonary infection. She subsequently died of sepsis on postoperative day 22.

## 3. Discussion

 Sciatic hernia is an extremely rare diagnosis. Pelvic floor hernias can be obturator, perineal, or sciatic, but the last is the rarest. Some comorbidities were described together with the cases of sciatic hernia reported in the literature, including neoplasms, coexisting hernias, congenital anomalies, pelvic bones disorders, metabolic diseases, pregnancy or multiparity, and malnourishment [1]. Although cirrhotic patients frequently develop hernias, this is the first report of a case of sciatic hernia in such patients.

 Cirrhotic patients are immunosuppressed by their condition. Moreover, the perianal region is a frequent site of infection and abscess in the general population and in patients with chronic liver diseases. Combined with the rarity of a sciatic hernia, the frequency of perianal infection may have been the reason for the misdiagnosis. Generally, abdominal hernias are easy to diagnose in physical examination, but when there is some doubt or need to evaluate better, a CT scan or magnetic resonance should be done. In this particular case, the outcome may have been better if the first doctor asked for a CT scan before the first surgical procedure. For that reason, doctors have to be aware that cirrhotic patients can develop uncommon hernias and deserve image studies before undergoing surgical or invasive procedures. 

 The surgical procedure for the correction of this hernia has not been standardized, and abdominal, gluteal, and combined approaches have been described. Moreover, a laparoscopic approach was used in 21% of cases [1, 3, 4]. The transabdominal is preferred when there is a suspicion of small bowel incarceration or strangulation [5]. Besides the small bowel, in patients without liver diseases, the hernia may contain ovary, bladder, ureter, colon, or appendix [3]. On the other hand, in cases of cirrhotic patients, the content is mostly ascites [6]. In these cases, the transgluteal approach is preferred because it is less invasive and is easier to obtain a hermetic closure. In cirrhotic patients, the main concern is ascites leakage, and impermeability of the surgical site is mandatory. Indeed, a sciatic hernia is a difficult diagnosis. However, cirrhotic patients have an abdominal wall and pelvic floor weakness that is associated with ascites pressure. As such, these patients develop common abdominal hernias, such as umbilical and inguinal hernias, and uncommon hernias, such as pelvic floor hernias [2].

 In fact, all kinds of hernias in advance cirrhosis are difficult to manage. These patients easily decompensate and get infection, develop renal insufficiency, or worsen their liver function. The morbidity and mortality seem to be higher in urgent situations, with hernia perforation or incarceration and when infection is associated [6]. The surgical technique needs to be adapted prioritizing the hermetic closure. In summary, elective surgery is the treatment of choice, and such patients are better treated in reference centers, with transplant and hepatobiliary surgeons, where there are experience with surgery in cirrhotic patients, clinical management in postoperative period, and available liver transplant. 

## Abbreviation

MELD: Model for end-stage liver disease.

## References

[1] Losanoff JE, Basson MD, Gruber SA, Weaver DW (2010). Sciatic hernia: a comprehensive review of the world literature (1900–2008). *American Journal of Surgery*.

[2] Andraus W, Sepulveda A, Pinheiro RSN, Teixeira AR, D’Albuquerque LAC (2010). Management of uncommon hernias in cirrhotic patients. *Transplantation Proceedings*.

[3] Bernard AC, Lee C, Hoskins J (2010). Sciatic hernia: laparoscopic transabdominal extraperitoneal repair with plug and patch. *Hernia*.

[4] Witney-Smith C, Undre S, Salter V, Al-Akraa M (2007). An unusual case of a ureteric hernia into the sciatic foramen causing urinary sepsis: successfully treated laparoscopically. *Annals of the Royal College of Surgeons of England*.

[5] Servant CTJ (1998). An unusual cause of sciatica: a case report. *Spine*.

[6] Silva FD, Andraus W, Pinheiro RS (2012). Abdominal and inguinal hernia in cirrhotic patients: what's the best approach?. *Arquivos Brasileiros de Cirurgia Digestiva*.

